# Picture a scientist: classification images of scientists are perceived as White, male, and socially inept

**DOI:** 10.3389/fpsyg.2025.1575123

**Published:** 2025-04-30

**Authors:** Maheen Shakil, Hasan Siddiqui, M. D. Rutherford

**Affiliations:** Department of Psychology, Neuroscience and Behaviour, McMaster University, Hamilton, ON, Canada

**Keywords:** stereotypes, stem, social representations, face perception, classification images

## Abstract

**Introduction:**

Stereotypes and biases toward social categories are often reflected in mental representations of faces. The current study used a two-phase reverse correlation procedure to visualize mental representations of the faces of a scientist, a hero, a genius, and a person.

**Methods:**

In the first phase, 20 participants completed four blocks of a two-image forced-choice task. In each block, they selected which face from a pair resembled one of the four categories. The images they selected were averaged to create classification images (CIs), which serve as proxy images for their mental representations of the four categories. In the second phase of the study, 251 naive participants rated the CIs based on various valence and demographic characteristics.

**Results:**

We found that the scientist image was rated predominantly as White and male, which reflects stereotypes about who pursues scientific careers. The scientist image was also rated more negatively than the other CIs on several characteristics, which may indicate negative biases toward scientists as unsociable, poor communicators, and incompetent authority figures, especially during the COVID-19 pandemic.

**Discussion:**

These findings deepen our understanding of how social categories are represented and demonstrate how the CI method can reveal stereotypes and attitudes related to these social categories.

## Introduction

Picture a scientist. What kind of person do you envision? Your mental image of a scientist reflects stereotypes associated with the profession, including sex, race, and age. For the last five decades, developmental psychologists have employed “draw a scientist” tests to assess children’s stereotypes of scientists. Although gender diversity has increased somewhat over the decades, most children draw a White man when asked to draw a scientist (Miller et al., 2018). This bias likely reflects the children’s observations: Historically, scientists have mostly been represented as White men (Watts, 2007). In North America, women and minorities have struggled to make a place in science and be recognized for their work (Campbell et al., 2000). While the proportion of women and other minorities in the sciences has increased, the majority of scientists are still White and male. In the United States, as of 2019, men made up 66% of the workforce in Science, Technology, Engineering, and Mathematics (STEM), compared to 48% in non-STEM fields. In the United States, White workers constitute 64% of the STEM workforce in the United States compared to the 61% share of the overall workforce (National Science Board, National Science Foundation, 2021).

Even when women manage to enter the scientific workforce, they face challenges. Men publish more papers, attain more senior positions in academia, and apply for more grants than women (Huang et al., 2020; Larivière et al., 2013; Ley and Hamilton, 2008). Several explanations have been proposed for this disparity, such as parental care, biases in review processes, resource allocation, and work culture (Borsuk et al., 2009; Bronstein and Farnsworth, 1998; Cameron et al., 2016; Duch et al., 2012; Stack, 2004). The stereotypes about scientists likely exacerbate the obstacles that women and minorities encounter in the sciences, as well as the challenges when entering the field. The 2020 documentary *Picture a Scientist* recounted the experiences of three female scientists and the challenges they faced as women in STEM (Shattuck and Cheney, 2020). Women’s struggles to achieve in science can, in turn, reinforce stereotypes of scientists.

One way to understand stereotypes is by visualizing people’s mental representation of a social category of interest, like the category of scientists. Mental representations are shaped through experience with a given category (Valentine et al., 2004). It is theorized that individuals identify each other’s social category membership by matching their faces to their mental representations of those social categories (Freeman and Ambady, 2014). This process occurs rapidly for social categories, such as race, gender, and age (Ito and Urland, 2003, 2005; Mouchetant-Rostaing et al., 2000; Tanaka, 2001). Individuals also categorize others based on categories without clear perceptual markers, such as political affiliation, social class, and religious identity, more accurately than would be expected by chance (Andrzejewski et al., 2009; Bjornsdottir and Rule, 2017; Olivola et al., 2012; Rule and Ambady, 2010). This implies that individuals maintain unique mental representations for these categories, and occupation may also be one of them. This rapid process of social categorization not only activates mental representations for a specific social category but also triggers the associated stereotype content associated with them (Freeman and Ambady, 2009; Hugenberg and Bodenhausen, 2003). The immediate impressions formed about an individual’s face have significant consequences in the real world, including employment, political, financial, and judicial outcomes (Rule et al., 2016; Olivola and Todorov, 2010; Rule and Ambady, 2011; Todorov et al., 2005; Zebrowitz and McDonald, 1991). The stereotypes individuals hold about scientists, regarding their demographic traits and their other characteristics, may be reflected in their mental representations. The reverse correlation method allows us to create an image that depicts someone’s mental representation of a scientist.

Reverse correlation (RC) is a technique used to create a visual representation of mental representations, particularly of different faces (Brinkman et al., 2017). Randomly generated noise patterns are superimposed over face images to create large sets of varied stimuli, and participants rate or select the stimuli that most resemble the category of interest. For instance, when visualizing female face representations, participants might choose which of two noise-altered faces looks more female, or rate how female a given noise-altered face looks. These selections are then averaged to create a classification image (CI), which serves as a proxy for their mental representation of female faces. Reverse correlation has been used to visualize representations of faces of different genders, occupations, ethnicities, religions, degrees of sickness, and more (Brown-Iannuzzi et al., 2018; Dotsch et al., 2008; Dunham et al., 2014; Hehman et al., 2015; Imhoff et al., 2013; Mangini and Biederman, 2004; Ojeda et al., 2022). It has also been used to visualize bodies, and objects such as cars (Diego-Mas et al., 2022; Lick et al., 2013; Maister et al., 2021).

Many RC studies will incorporate a rating task in which the CIs are rated by a naive sample of participants based on various traits of interest. These ratings can reveal information about stereotypes and attitudes toward the categories represented by the CIs. The biases the first participant group had toward the categories of interest are reflected in the CIs their image selections created, and the ratings from the second set of participants are meant to reveal those biases. The advantage of the RC method is its ability to implicitly assess attitudes toward social categories. Studies using this paradigm have shown, for instance, that individuals with implicit bias against Moroccans visualize faces generated to represent Moroccans as criminal and untrustworthy, and that atheists are perceived negatively compared to theists (Brown-Iannuzzi et al., 2018; Dotsch et al., 2008). CIs can also reflect an individual’s assumptions of how members of a social group will behave. For instance, CIs representing theists are expected to behave more morally than those representing atheists (Brown-Iannuzzi et al., 2018). This suggests that mental images can store stereotypes and also reinforce them. Visualizing how people mentally represent scientists can thus shed light on the stereotypes associated with them, especially those that individuals may be unaware of or reluctant to endorse due to social desirability bias (Tourangeau and Yan, 2007).

While RC has been used to study a variety of social categories, it has not been applied to the study of the category of scientists. It is interesting to examine how stereotypes of scientists differ from those of other categories. Scientists are characterized as intelligent, as are “geniuses.” Unlike scientists, geniuses may be more likely to be perceived as innately gifted and may be more easily associated with any gender or ethnicity. Unlike scientists, they are also not necessarily professionals–one can be a genius in any number of areas. Scientists’ roles as professionals can also be the basis for distrust. They may be seen as out of touch with the general population, occupied by their work in their “ivory tower” (Locke, 1999; Stilgoe et al., 2014). The longstanding issues in the realm of scientific communication can also exacerbate this perception (Koswatta et al., 2023). A “hero” and a scientist may possess traits such as competence and helpfulness, but they may contrast on other traits due to the specific nature of the scientist label. A hero would not have the same training, and could be labeled a hero based on a unique, spontaneous event, such as saving a child from a river. Heroes are also more likely to be seen as altruistic and kind, while scientists are often stereotyped as cold and aloof (Fiske and Dupree, 2014; Fujiwara et al., 2022). Determining whether scientists, geniuses, and heroes have distinct mental face templates would expand our understanding of the types of categories that are represented distinctly in the mind.

Finally, another category which should have a corresponding mental representation is a superordinate one: “person.” Most RC studies select specific social categories, such as gender, ethnicities, and profession, and examine their mental representations. To our knowledge, no one has yet investigated how individuals generally represent a person. This representation would also serve as an interesting contrast to categories like scientist, genius, and hero, as a person should encompass all possible traits, while the others might have particular characteristics based on stereotypes.

## The current study

The purpose of the current study is to employ an RC paradigm to visualize mental representations of a scientist, and compare these representations to those of a genius, a hero, and a person. In the first phase of the study, participants selected face images in a two-image forced-choice task that resembled each of the four categories being tested. The stimuli they selected were averaged to create CIs, which were then rated on several valence and demographic characteristics by a naive set of participants. These ratings revealed whether these four categories are viewed positively or negatively, and which traits differed across categories. Ratings also revealed whether these categories are associated with specific genders and ethnicities, highlighting any demographic biases and stereotypes that may exist within these categories. We predicted that the Scientist CI would be rated as White and male in contrast to the other CIs. We also predicted that it would have similar ratings to the genius for traits related to intelligence, and to the hero for traits related to competence. The CI for the category of person was expected to be neutral on valence and demographic traits, as it represents a superordinate category.

## Phase 1: image selection

### Methods

#### Participants

Twenty participants (mean age = 18.35 years, 4 males) were recruited for the first phase of the experiment. This sample size was based on sample sizes reported in past reverse correlation research (e.g., Dotsch and Todorov, 2012). Participants were undergraduate students at McMaster University and received course credit for participation. Five participants identified as South Asian, five as East Asian, four as White, three as South East Asian, one as Black, and one as mixed-race (South Asian and East Asian).

#### Procedure

##### Stimulus creation

A base image was created by averaging 30 neutral faces (15 males and 15 females) from the four ethnic groups, White, Black, Asian, and Latinx. The faces were taken from the Chicago Face Database (Ma et al., 2015). The average was generated in Webmorph, the web-based version of Psychomorph (DeBruine, 2018). Randomly generated sinusoidal noise patterns were overlaid on the base image using the *generateStimuli2IFC* function from the *rcicr* package in R version 4.2.1. Three hundred stimulus pairs were created, with each pair consisting of the base image with a randomly generated noise pattern and the base image with the inverse noise pattern overlaid, such that the dark pixels in one image corresponded to light pixels in the other.

##### Image selection

Participants completed four blocks of a two-image, forced-choice, reverse correlation task. In each block, participants were asked to select, by clicking on one of the images, the face that most resembled the category label. In one block, they selected the face that looked like a scientist. In another block, they selected the face that resembled a hero, in another, the face that resembled a genius, and in another, the face that resembled a person. Each block consisted of 300 trials, presented in a randomized order for each participant. The order of the blocks was randomized for each participant.

##### Demographic questionnaire

Participants were asked to report their age, gender, ethnicity, nationality, religious identity, and socioeconomic status prior to the study.

### Results

#### Classification image creation

**Figure 1:**
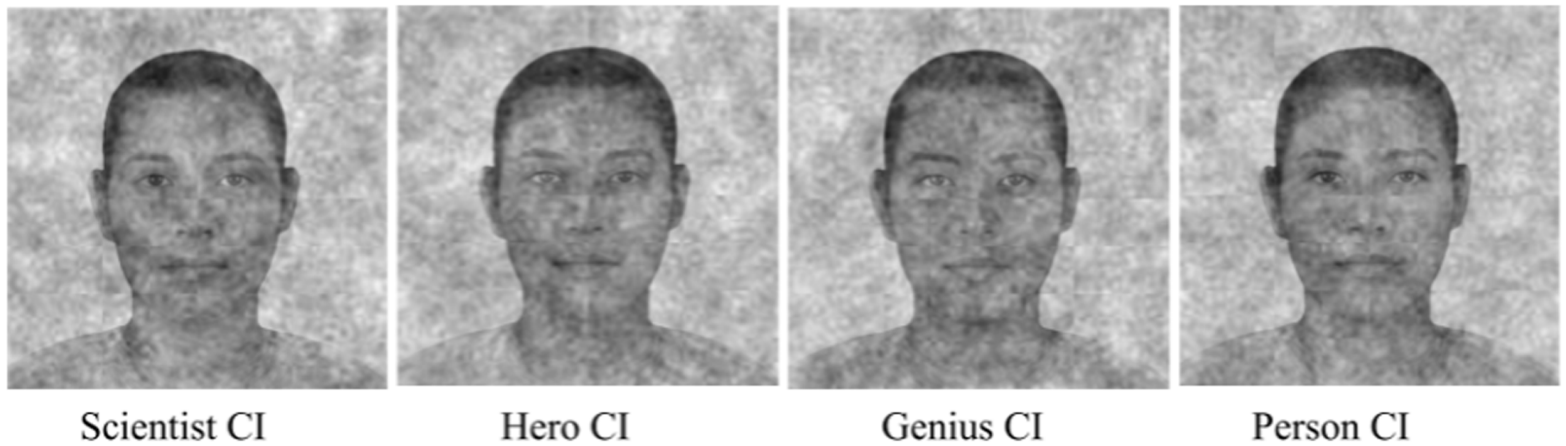
The 4 classification images (CIs) that were created using data from phase 1. The base images used to create these CIs were formed by averaging faces from the Chicago Face Database (Ma et al., 2015).

The images selected in each of the four blocks were averaged using the *generateCI2IFC* function from the *rcicr* package in R version 4.2.1 (Brinkman et al., 2017). The resulting averages for each block were the four Classification Images (CIs), and these are taken to be proxy images for mental representations of the categories of interest. Thus, this process yielded CIs that represent what a scientist, hero, genius, and person look like (see Figure 1).

## Phase 2: image rating

### Methods

#### Participants

A total of 251 participants (mean age = 35.04 years, 136 males) were recruited from Amazon MTurk and were paid 4.60 Canadian dollars (CAD) as compensation for their participation in accordance with Ontario’s minimum wage. A sample size of 251 was deemed necessary to have 80% power to detect differences in CI ratings across groups, based on the effect size reported in the Supplementary material of the study by Dunham et al. (2014). The power analysis was conducted using G*power 3.1. In terms of ethnicity, 154 participants identified as White, 33 as South Asian, 27 as East Asian, 10 as Middle Eastern, 7 as South East Asian, 4 as Latinx, 4 as Black, 2 as Indigenous North American, and 6 identified as mixed-race (2 White and East Asian, 1 White and South Asian, 1 White and Latinx, 1 White and Black, and 1 unspecified).

#### Selecting valence characteristics

One block of the rating task involved rating the CIs from phase 1 on valence characteristics. We were interested in using valence ratings that examined characteristics relevant to scientists and could reveal potential gender biases. To do this, we gathered data from Ben Schmidt’s “Gendered Language in Teaching Evaluations” database on 12 January 2022 (Schmidt, 2015). This database collects and displays terms used to describe professors on Ratemyprofessor.com, which is categorized by gender. For our purposes, we retrieved terms that were used to describe professors in the sciences. We selected terms with the most significant disparity in how they were used to describe male vs. female professors. Ten male-gendered terms and nine female-gendered terms were selected, and antonyms for each term were generated to use as labels for the rating scale endpoints. The term “clear,” which was taken from the database, was changed to “well-spoken” to prevent participants from rating the image on visual clarity. The terms are shown in Table 1.

**Table 1:** Traits selected for valence ratings in phase 2 of the study, with their frequencies (in words per million) and male-to-female ratios.

Trait	Male frequency	Female frequency	Male-to-female ratio	Female-to-male ratio
Arrogant	119.9	35.4	3.39	0.30
Charismatic	9.6	3.6	2.67	0.38
Entertaining	252	113	2.23	0.45
Funny	1,538	719	2.14	0.47
Brilliant	117	69	1.70	0.59
Cool	581	350	1.66	0.60
Chill	37	25	1.48	0.68
Smart	517	355	1.46	0.69
Engaging	90	65	1.38	0.72
Intelligent	256	186	1.38	0.73
Clear	977	1,150	0.85	1.18
Nice	2,285	2,702	0.85	1.18
Personable	48	57	0.84	1.19
Competent	9.06	11.15	0.81	1.23
Mean	186	233	0.80	1.25
Professional	51	64	0.80	1.25
Organized	208	299	0.70	1.44
Strict	74	112	0.66	1.51
Warm	11.3	18.7	0.60	1.65
Sweet	126	342	0.37	2.7

Traits were collected from data for science professors in the Gendered Language in Teaching Evaluations Database.

#### Procedure

##### Rating tasks

Participants completed two blocks of a rating task. In the first block, participants rated the four CIs on 19 valence characteristics. In the second block, participants rated the four CIs on 10 demographic characteristics, including gender and ethnicity. The demographic characteristics were male, female, White, Black, Latinx, East Asian, South Asian, Middle Eastern, Pacific Islander, and Indigenous Canadian. The two blocks were presented in this fixed order to prevent social desirability effects on responses. During the rating task, participants viewed one of the CIs on the computer screen, accompanied by a slider representing a 6-point Likert scale below it. Participants clicked on the slider to indicate how much the CI fit the characteristic. Each participant rated all four CIs on all of the characteristics. The trials within each block were presented in a randomized order across participants; however, the order of the blocks was arranged so that all participants completed the valence ratings before the demographic ratings.

##### Demographic questionnaire

Participants in phase 2 of the experiment completed the same demographic questionnaire as those in phase 1.

#### Data availability statement

All study materials and raw data for both phases of this experiment are openly available on OSF at https://osf.io/ycnzg/?view_only=57a646052c2d49fcb0b948355b10b562.

### Results

#### Findings: demographic traits

**Figure 2:**
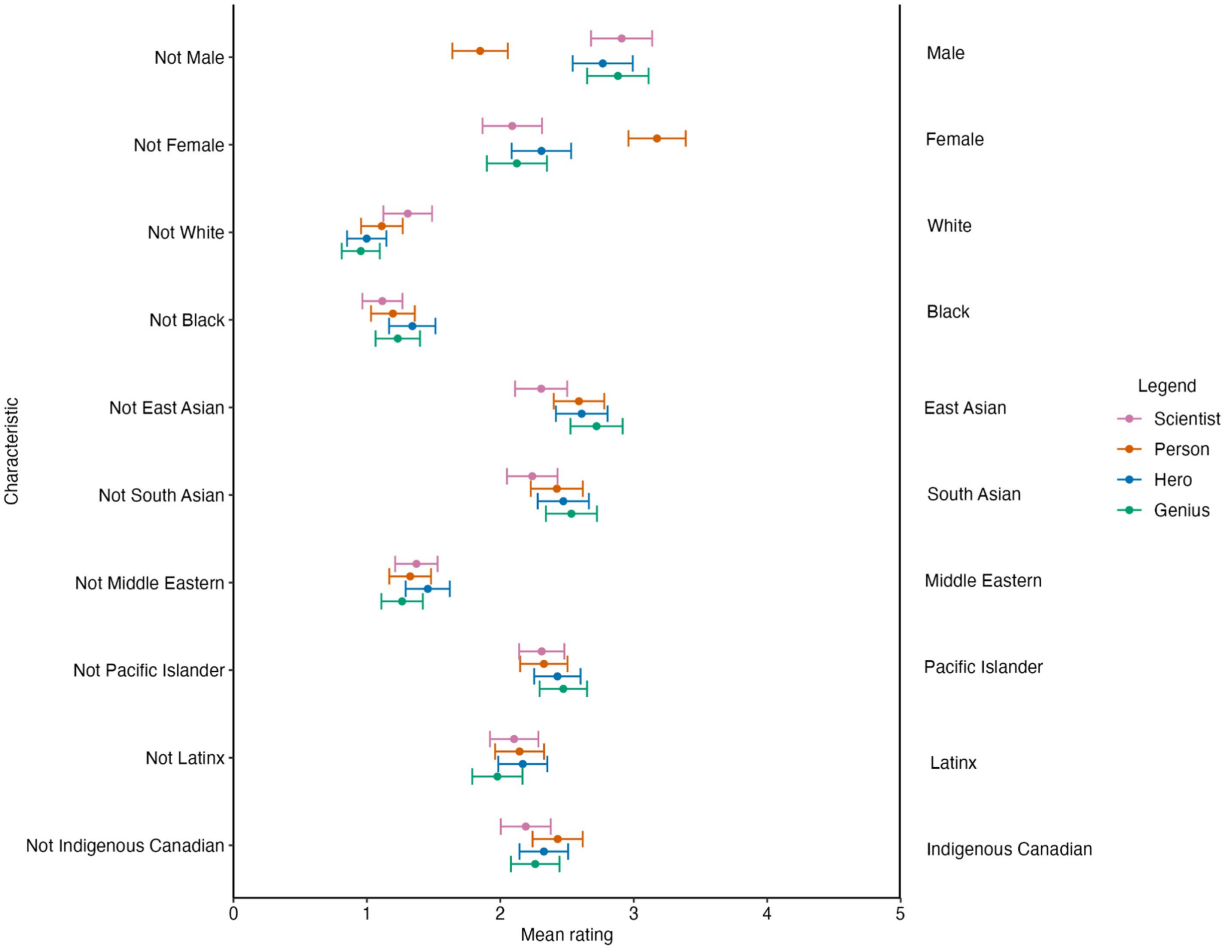
Mean ratings of confidence intervals (CIs) on demographic traits. Error bars represent 95% CIs.

We conducted a within-subjects analysis of variance (ANOVA) using rating scores as the dependent variable^1^. Independent variables included classification image (CI; scientist vs. hero vs. person vs. genius) and trait (10 levels, see Figure 2). We found a significant main effect of CI (*F* (3, 738) = 5.72, *p* < 0.001, Cohen’s *f* = 0.14, 95% confidence interval CI [0.07, 0.22]). We also found a main effect of trait (*F* (9, 2,214) = 72.54, *p* < 0.001, Cohen’s *f* = 0.54, 95% CI [0.49, 0.58]). Critically, we found a significant CI × Trait interaction (*F*(27, 6,642) = 22.09, *p* < 0.001, Cohen’s *f* = 0.29, 95% CI [0.27, 0.32]).

At this stage, we conducted one-way within-subject ANOVA tests using CI as the within-subject independent variable. We found a significant effect of CI on “Female” Ratings (*F*(3, 738) = 54.85, *p* < 0.001, Cohen’s *f* = 0.47, 95% CI [0.39, 0.54]). *Post hoc* tests revealed that the person CI was rated as significantly more female than the remaining CIs (*p*-values below 0.001). We also found a significant effect of CI on “male” ratings (*F* (3, 738) = 58.47, *p* < 0.001, Cohen’s *f* = 0.48, 95% CI [0.41, 0.56]). *Post hoc* tests revealed that the person CI was rated as significantly less male than the other CIs (*p*-values below 0.001). Next, we examined the effect of CI on race demographic ratings. We found a significant effect of CI on “White” ratings (*F* (3, 738) = 11.05, *p* < 0.001, Cohen’s *f* = 0.20, 95% CI [0.13, 0.28]). Conducting *post hoc* analyses, the scientist CI was rated as significantly more White than the remaining CIs (*p*-values below 0.05). We also conducted a one-way ANOVA investigating the effect of CI on Black ratings. We found a significant effect of CI on Black ratings (*F* (3, 738) = 3.81, *p* = 0.01, Cohen’s *f* = 0.11, 95% CI [0.03, 0.19]). For Black ratings, the scientist CI was rated significantly less Black than the hero CI (*p* = 0.02); however, no other *post hoc* comparisons reached significance.

#### Findings: valence traits

As with the demographic traits, we first conducted a within-subjects ANOVA on rating scores with CI (scientist vs. hero vs. person vs. genius) and trait (19 levels, see Table 1) as independent variables, as well as the interaction between them. We found a significant main effect of CI (*F* (3, 738) = 52.99, *p* < 0.001, Cohen’s *f* = 0.46, 95% CI [0.38, 0.54]). *Post hoc* analyses revealed that the scientist CI received significantly lower scores (which were rated more negatively) than the other CIs (*p*-values below 0.001; see Figure 2). We also found a significant main effect of trait (*F*(18, 4,428) = 45.05, *p* < 0.001, Cohen’s *f* = 0.42, 95% CI [0.39, 0.45]). Critically, we again found a significant CI × Trait interaction (*F* (54, 13,284) = 6.62, *p* < 0.001, Cohen’s *f* = 0.15, 95% CI [0.13, 0.17]).

At this stage, we conducted one-way within-subjects ANOVAs with CI as the independent variable. All one-way within-subjects ANOVAs revealed a significant main effect of CI, but we report only the findings where the corresponding *post hoc* comparisons were significant after a Bonferroni correction. We found a significant effect of CI on “warm ratings” (*F* (3, 738) = 17.24, *p* < 0.001). The hero CI was rated as “more warm” (*p*-values below 0.005) than the remaining CIs, while the person CI was rated as “more warm” than the scientist. We found a significant effect of CI on “sweet/harsh” ratings (*F* (3, 738) = 15.17, *p* < 0.001, Cohen’s *f* = 0.24, 95% CI [0.17, 0.32]). Again, the hero was rated as “more sweet” (*p*-values below 0.005) than the remaining CIs. We found a significant effect of CI on “strict/lenient” (*F* (3, 738) = 6.34, *p* < 0.001, Cohen’s *f* = 0.15, 95% CI [0.08, 0.23]) and “mean/king” (*F* (3, 738) = 7.79, *p* < 0.001, Cohen’s *f* = 0.17, 95% CI [0.09, 0.24]) ratings. In this case, the hero CI was rated as “less strict” (*p*-values below 0.05) than the remaining CIs, and the scientist was “more mean” than the hero CI (*p* < 0.001).

There were various characteristics where the scientist CI was rated the lowest among the four CIs. In the cases of “funny/dull” (*F* (3, 738) = 23.54, *p* < 0.001, *f* = 0.31), “engaging/tedious” (*F* (3, 738) = 19.78, *p* < 0.001, *f* = 0.28), “cool/awkward” (*F*(3, 738) = 11.33, *p* < 0.001, *f* = 0.21), and “brilliant/dim” (*F* (3, 738) = 10.50, *p* < 0.001, *f* = 0.20) ratings, all CIs were rated more positively than the scientist CI (*p*-values below 0.001). Similarly, all CIs were rated as “more entertaining” (*F* (3, 738) = 14.02, *p* < 0.001, *f* = 0.24) and more Chill (less uptight) (*F* (3, 738), = 13.78, *p* < 0.001, *f* = 0.24) than the scientist (*p*-values below 0.002). The scientist was also rated as the lowest on “charismatic/uncharismatic” ratings (*F* (3, 738) = 10.81, *p* < 0.001, *f* = 0.21); specifically, they were rated significantly worse than the person and the hero CI (*p*-values below 0.001). The scientist was also rated as the lowest on competent/incompetent ratings (*F* (3, 738) = 8.81, *p* < 0.001, *f* = 0.19); specifically, the scientist was rated as less competent than the genius and person (*p*-values below 0.002). The scientist was also rated the lowest on smart/dumb ratings (*F* (3, 738) = 8.80, *p* < 0.001, *f* = 0.19); specifically, the scientist was rated as “less smart” than the genius and the person CIs (*p*-values below 0.001). Again, the scientist was rated lowest on the “personable/irritating” ratings (*F* (3, 738) = 6.29, *p* < 0.001, *f* = 0.16); specifically, the scientist was rated lower than the genius and the hero (*p*-values below 0.01). Finally, the scientist was rated as less intelligent (more obtuse) (*F* (3, 738) = 8.05, *p* < 0.001, *f* = 0.18) and “less well-spoken” (more ineloquent) (*F* (3, 738) = 7.38, *p* < 0.001, *f* = 0.17) than the person (*p* < 0.001).

#### Correlational analysis

For each of our valence traits, except for “well-spoken” (a term we created), we calculated the ratio of usage to describe male professors compared to female professors in reviews on RatemyProfessor.com. Higher ratios mean that the word was used more often to describe male professors than female professors. For example, the word arrogant had a ratio value of 3.38, meaning that the word arrogant is used 3.38 times more often to describe male professors than female professors. We correlated these ratios with the corresponding mean rating for each valence trait for the scientist CI. There was a significant negative correlation between the male-to-female ratio and mean responses (*r* (18) = −0.57, 95% CI [−0.82, −0.14], *p* = 0.01), indicating that more positive ratings were associated with more masculine traits.

#### Order effect

**Figure 3:**
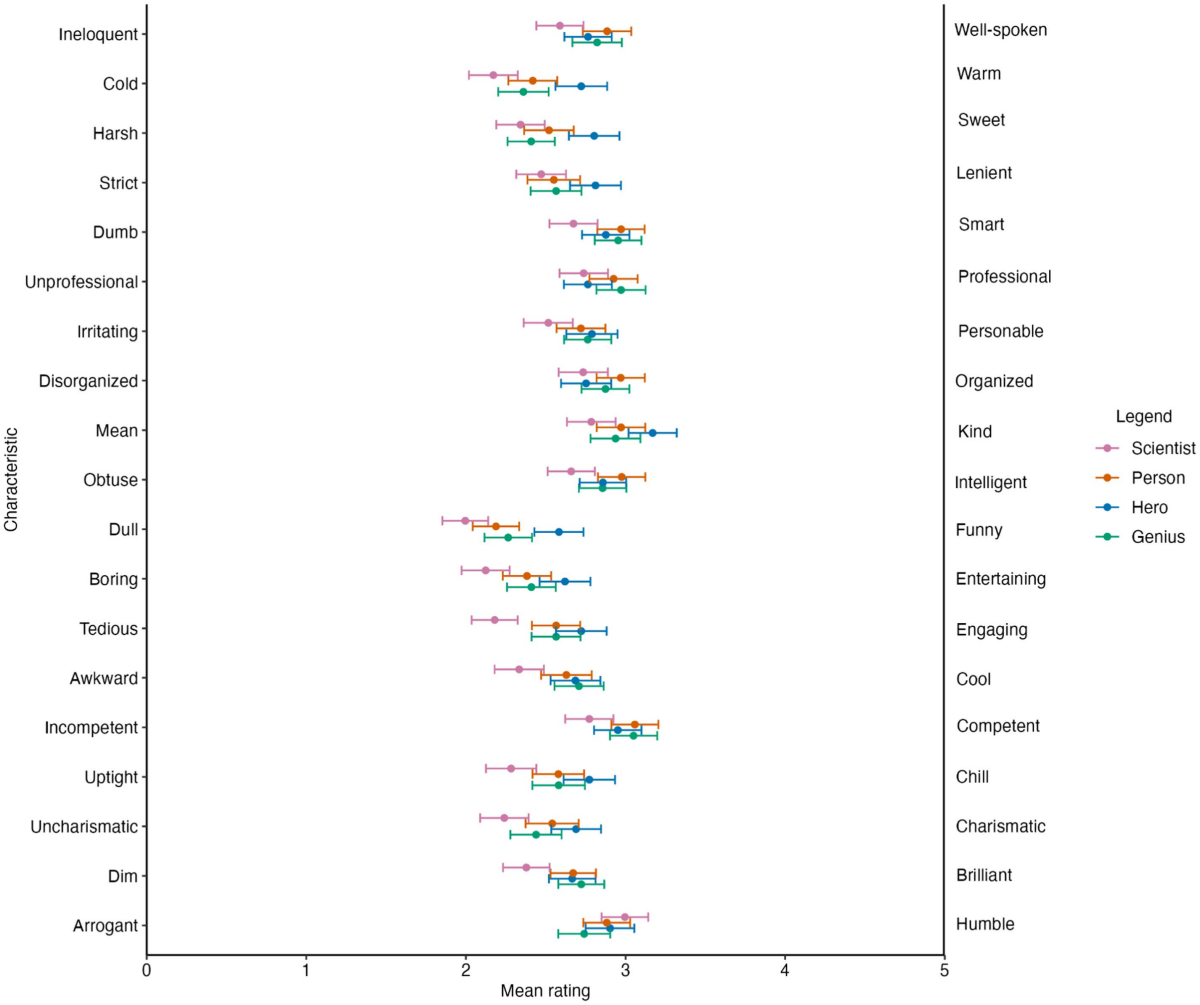
Mean ratings of confidence intervals (CIs) on valence traits. Error bars represent 95% CIs.

When graphically observing the data (see Figure 3), it appeared that the scientist CI was consistently rated the lowest across all personality traits. To assess if this was true, we conducted a chi-square test of independence to test the null hypothesis that there was no relationship between classification image and valence ranking. A significant chi-square test would indicate that certain CIs received the same ranking (e.g., highest, lowest, etc.) across multiple characteristics. We operationalized high and low mean ratings by “ranking” the mean ratings. Figure 3 shows the four confidence intervals whose means were visualized. If the mean rating for a CI was the highest among the four CIs for a given trait (e.g., happiness), it was counted as “first,” and that CI received one observation for the “first” rank. We recorded the frequencies at which the CIs were first, second, third, and fourth highest on the 19 valence characteristics listed in Table 2. The chi-square test was significant (*χ*^2^(9) = 75.58, *p* < 0.001). The scientist CI was ranked the lowest more often than would be expected by chance.

**Table 2:** Observed frequencies for CI means in each rank position for valence traits.

Classification image	Fourth	Third	Second	First
Scientist	18	0	0	1
Person	0	7	7	5
Hero	0	6	3	10
Genius	1	6	9	3

## Discussion

The purpose of this study was to visualize participants’ mental representations of scientists, heroes, geniuses, and other individuals, and to test whether demographic and valence characteristics were associated with these mental representations. Using a reverse correlation paradigm, we created images representing each category and collected demographic and valence ratings of each image. We found that the scientist image was rated as more male and more White than the other three categories, and had the most negative ratings overall. Specifically, the scientist was rated lower on traits associated with sociability, communication skills, and competence. The hero image was rated as warm and kind, and the person was rated as the most feminine in appearance.

The generation of four unique CIs in this study illustrates the utility of the reverse correlation method. We were able to create face images that reflect the mental representations of our participants. Further, we have extended previous research that has used reverse correlation to visualize individuals with certain occupations (Hehman et al., 2015; Imhoff et al., 2013). Similar to these studies, we found that people have representations of faces associated with specific occupations, even though occupation is a flexible, chosen identity. Since stereotypes about occupations are reflected in these mental representations, reverse correlation can be used to reveal stereotypes. This is especially useful given that reverse correlation is an implicit method—this allows us to capture more spontaneous responses, with less response biases than are present in methods that directly ask about stereotypes. Heroes and geniuses are even more nebulous categories than scientists, but participants revealed distinct representations that reflect stereotypes for these categories as well. The reverse correlation method lends itself to exploring attitudes and stereotypes about various social categories and labels that have not been examined before.

The fact that the Scientist CI was perceived as more male and more White than other categories suggests that there is a stereotypical image of a scientist, and this image is relatively White and male. This result is consistent with the idea that mental representations of faces are based on all of the faces one has encountered. In the United States, White males traditionally pursue careers in the sciences more than women or people of color do (Campbell et al., 2000). This would lead to the formation of a White, masculine mental representation of a scientist, as it reflects the characteristics of the scientists that people have seen.

The way scientists are represented in the media may also contribute to people’s mental representations. News stories about scientists differ by gender. While typically masculine and feminine traits (e.g., drive and collaboration, respectively) are attributed to scientists of all genders, the personal lives and work–life balance of female scientists receive more attention (Mitchell and McKinnon, 2019).This perpetuates the idea that science is something men can perform without having to balance anything else, while women are expected to balance priorities.

The scientist, hero, and genius were all rated as more masculine in appearance than the person. As discussed above, men outnumber women in the sciences. Heroes may also be male-coded: Fictional male superheroes often outnumber their female counterparts and tend to exhibit stereotypically masculine traits, whereas female superheroes are often feminine and sexualized (Anderson and Cavallaro, 2002; Cocca, 2014; Harriger et al., 2022). Geniuses may be perceived as more male due to the associations between intelligence and maleness. Intelligence, in the West, is conceptualized as a rational, logical trait, which is considered to be a masculine trait (Räty and Snellman, 1992; Sternberg, 1985; Sternberg et al., 1981). Compared to these three categories, the superordinate category of person would appear less masculine.

The scientist CI consistently received the least favorable ratings of any of the four CIs across all valence characteristics. Specifically, it was rated significantly lower than other CIs on characteristics that can be grouped into three “themes,” which reflect perceptions individuals have of scientists. First, the scientist CI was perceived as less sociable and was rated significantly as more dull, tedious, awkward, and irritating. The scientist CI was seen as a poor communicator, and was rated less engaging, charismatic, entertaining, and well-spoken. Finally, the scientist CI was even seen as less capable academically, and was rated less brilliant, competent, smart, and intelligent. Some of these findings reflect broad stereotypes about scientists, and others can be understood by comparing the connotations of being a scientist, vs. a hero, genius, or a regular person. When compared to the hero and genius, the authority associated with the role of a scientist might explain their relatively negative perception. Heroes, by definition, are altruistic and use their abilities for the greater good. Scientists do not necessarily do so, and their portrayals in fiction perpetuate this idea. In contrast, scientists and geniuses share the connotation of intelligence and competence, but scientists are professionals, often in roles of authority. Distrust toward authorities and attitudes toward scientific guidance on issues like climate change, vaccines, and, more recently, the COVID-19 pandemic, might mean scientists are viewed as less competent and poorer at communicating.

Heroes are perceived as more powerful and competent than the general population, and this allows them to save others and lead them through difficulties (Kinsella et al., 2015a, 2015b). They are seen as altruistic and likeable (Fradkin et al., 2016; Muller et al., 2020). This applies to heroes in real life (e.g., Volodymyr Zelenskyy’s leadership during the invasion of Ukraine) as well as in fiction. In theory, scientists are also considered competent in their area of expertise, distinguishing from the general population. These two categories can overlap, as some scientists are recognized as heroes for their groundbreaking contributions to science and the betterment of society, such as Alan Turing’s code-breaking in World War II and Katherine Johnson’s contributions to NASA’s first human-crewed space flight (Bullynck et al., 2015; Malcom, 2020). However, not all scientists are viewed this way.

The hero was rated as warmer and kinder than the scientist, and the scientist was also rated lower on other traits associated with sociability, perhaps due to the stereotypical image of scientists as cold, clinical, and calculating (Fiske and Dupree, 2014; Fujiwara et al., 2022). While the ability to be objective and thorough is helpful for research, it does not make scientists appear approachable or likable. The Western conception of logical intelligence being separate from social and emotional intelligence may also feed the perception of scientists as logical individuals who are not socially adept (Sternberg, 1985; Sternberg et al., 1981). However, it is essential for scientists to gain not only the respect but also the trust of the general public by projecting warmth and competence, thereby effectively communicating and enacting their findings (Fiske and Dupree, 2014).

Unfortunately, distrust of scientists is prevalent, and appears to be increasing, especially regarding issues like climate change and vaccines, and this may have contributed to the surprising finding that individuals perceive scientists as incompetent (Dixon and Jones, 2015; Kabat, 2017; Peters, 2013; Pittinsky, 2015; Rutjens et al., 2018a; Rutjens et al., 2018b). Some of this lack of trust is related to individuals’ ideologies and political views, and whether these views align with those of scientific authorities. For instance, there is growing distrust among right-wing individuals in the United States toward scientists, due to their perception that scientific institutions are becoming increasingly liberal (Cofnas et al., 2018). This also applies to COVID-specific distrust of scientists, with right-wing individuals being less trusting of scientists and vaccine mandates than during the pandemic (Kossowska et al., 2021). The recommendations scientists made regarding social distancing, mask wearing, and vaccination were viewed by many as part of a larger agenda. This sentiment has only increased with the emergence of the COVID-19 pandemic, which coincided with the time this study was run (Sanchez and Dunning, 2021). During this time, scientists became an important authority, informing communication about and responding to the pandemic (Grundmann, 2021; Van Dooren and Noordegraaf, 2020). There were many who felt that scientists were overstating the risks associated with COVID-19 or that guidelines were infringing on their personal freedoms. Updates to guidelines could be perceived as mixed messages and may have exacerbated the view among the general public that scientists are incompetent and that they struggle to communicate information clearly. While scientific findings and guidelines are the products of rigorous study, the gap between the work and the public’s understanding of it, especially when information is presented differently by various outlets—scientists, journalists, governments, et cetera—can lead to the perception that scientists are out of touch, and do not know what they are doing (Salita, 2015; Weingart and Guenther, 2016). Negative messaging in the media also likely contributed to a lack of trust in scientists and their competence. News outlets in certain parts of the world portray scientists as members of the corrupt elite in contrast to the general population (Kulas, 2018). In the United States, President Donald Trump and right-wing media outlets downplayed the expertise of scientific authorities, such as the World Health Organization and leading American scientists, including Dr. Anthony Fauci (Raihl and Lilian, 2023; Rutledge, 2020). There is some research suggesting that President Trump’s messaging did not have a direct effect on individuals’ attitudes toward scientists and their competence in the United States (Evans and Hargittai, 2020). Instead, their social group membership—political alignment, race, religion, and socioeconomic status—seems to be a better predictor. However, it is important to note that this research was conducted using self-report measures, whereas the current study used an implicit measure. Even individuals who outwardly express belief in scientists’ competence may implicitly hold the opposite view, and this may explain our pattern of findings. While there are many possible explanations for feelings of distrust and negativity toward scientists, it is possible that the context of the COVID-19 pandemic contributed in some way to the sentiments reflected in the scientist CI and its ratings.

A significant amount of distrust in science comes from conspiracy-like beliefs, where individuals believe that others are secretly working toward nefarious ends (Douglas et al., 2017). In these cases, individuals fear that scientists are not acting objectively, and are instead working under the influence of some other power or corporation (Dixon and Jones, 2015). These sentiments likely increased negative sentiments toward scientists, leading individuals to select colder, less friendly-looking faces when completing the scientist image selection task. These attitudes may be exacerbated by real cases of unethical research, such as the Tuskegee Syphilis Study, where researchers studying the advancement of syphilis withheld life-saving treatment from the hundreds of Black men in the study (Brandt, 1978).

Fictional representations of scientists can also be negative. Consider the trope of the “mad scientist” (Weingart et al., 2003). It is very common for scientists to be portrayed as villains in fiction—consider characters such as Dr. Jekyll, Dr. Frankenstein, and the geneticists in *Jurassic Park*. Fictional scientific organizations include Aperture Science, featured in the *Portal* series of video games, and the Umbrella Corporation in the *Resident Evil* series of games. These scientists are typically shown experimenting, often in secret, with the human body and human nature, and creating things that are harmful to our health (Weingart et al., 2003). As such, even scientists who do their job well may be associated with different traits than heroes, geniuses, and ordinary people are.

There are caveats to consider when interpreting the results of this study. First, the negative results regarding scientists may be a result of this study’s context: the COVID-19 pandemic. Conducting a similar study at a different time, or with different populations, that have varying exposure to scientific information and scientific involvement in pandemic-related regulations might yield different results. Studying individuals who are not science undergraduate students or who have particularly strong feelings about science might also yield different results and would be an interesting area of study for the future research. Our sample may have also influenced the characteristics seen in the CIs: specifically, the person, which appeared more female than the other three CIs. Our sample consisted of more females than males, and they may have a more feminine representation of a typical person, perhaps because they have more interaction with other females. Future studies using undergraduate participants could also collect information about participants’ majors or recruit students from specific majors (e.g., science and non-science students) to see how their area of study influences their view of scientists.

The impacts of the base images on the resulting CIs are not quantified but are likely to be substantial. In our study, the base images used to create the stimuli included faces of males and females of various ethnicities. Many reverse correlation studies create their stimuli solely using Caucasian male faces (e.g., Dotsch et al., 2008). It is possible that the characteristics of a CI, especially its perceived gender and ethnicity, are constrained or influenced by the gender and ethnicity of the base images. Future studies could explore this issue and determine, if any, impact the starting point on the end product in a reverse correlation study.

The specific two-phase reverse correlation method also has certain caveats. Recent studies have shown that when CIs are created by aggregating the images selected by all participants, and those CIs are presented to new participants for rating, the differences between those CIs can be overestimated, and this can lead to an inflated type-I error rate (Cone et al., 2021). As such, it is possible that the findings in the current study were significant, or that the differences between CIs appeared larger due to the way the CIs were generated. To address this, we included effect sizes, with confidence intervals, for all main effects and interactions, and only reported on *post hoc* comparisons that remained significant after correction. Often, in cases where Type I error rates are likely, significant *p-*values are associated with weak effect sizes as indicated by confidence intervals that cross zero (e.g., Rothman, 2010). In our study, no effect sizes were associated with confidence intervals that crossed zero. This increases confidence that the effects observed in our study were not merely Type I errors (Rothman, 2010). Additionally, we included non-parametric analyses to support the conclusion that there were systematic differences in how CIs were rated. With this in mind, future studies could generate CIs for each participant in the first phase of the experiment and collect ratings, or calculate informational value of the CIs to assess stereotypes with a lower Type I error rate.

Finally, the current work may have benefited from the use of factor analysis to combine our valence traits into fewer factors. Doing so would have reduced the number of *post hoc* comparisons necessary across CIs. Due to the repeated-measure nature of our data, a *post hoc* exploratory factor analysis would not have been suitable (Newsom, 2023). Participants responded to the same valence question multiple times, and those responses could not be combined or correlated as they differed systematically by CI (Newsom, 2023). However, future research would benefit from *a priori* confirmatory factor analysis. Prior research using the Stereotype Content Model indicates that many of our valence traits may have combined into factors such as “warmth” or “competence” (see Cuddy et al., 2008). As such, future research using similar traits could utilize the Stereotype Content Model to support the theoretical development of factors that reduce the number of *post hoc* comparisons.

## Conclusion

This study aimed to visualize the mental representations of scientists, geniuses, heroes, and ordinary people, and to measure the demographic and valence traits associated with each category. We found that scientists are perceived as relatively White and male, reflecting stereotypes of who works in the sciences, as well as media and fictional portrayals that exaggerate the extent to which White men dominate science. Scientists are also rated low on characteristics associated with sociability, competence, and communication abilities. This may reflect negative stereotypes associated with scientists as authority figures, especially when they are the face of issues like the COVID-19 pandemic. Future research can explore other occupations, even within the sciences, to learn about the stereotypes and attitudes that are held toward these groups.

## Data Availability

The datasets presented in this study can be found in online repositories. The names of the repository/repositories and accession number(s) can be found at: https://osf.io/ycnzg/?view_only=57a646052c2d49fcb0b948355b10b562.

## Footnotes

^1^We conducted within-subject ANOVAs to mimic analyses conducted in previous reverse correlation studies using social categories (e.g., Dunham et al., 2014). However, to account for the nested nature of the data, mixed-effect linear models were conducted to mimic the analyses presented here. The results of the mixed-effects linear models can be found in Appendix A.

## Supplementary material

The Supplementary material for this article can befound online at: https://www.frontiersin.org/articles/10.3389/fpsyg.2025.1575123/full#supplementary-material

## References

[ref1] Anderson K. J. Cavallaro D. (2002). Parents or pop culture? Children’s heroes and role models. Child. Educ. 78, 161–168. doi: 10.1080/00094056.2002.10522728

[ref2] Andrzejewski S. A. Hall J. A. Salib E. R. (2009). Anti-Semitism and identification of Jewish group membership from photographs. J. Nonverbal Behav. 33, 47–58. doi: 10.1007/s10919-008-0060-z

[ref3] Bjornsdottir R. T. Rule N. O. (2017). The visibility of social class from facial cues. J. Pers. Soc. Psychol. 113, 530–546. doi: 10.1037/pspa0000091 28557470

[ref4] Borsuk R. M. Aarssen L. W. Budden A. E. Koricheva J. Leimu R. Tregenza T.. (2009). To name or not to name: the effect of changing author gender on peer review. Bioscience 59, 985–989. doi: 10.1525/bio.2009.59.11.10

[ref5] Brandt A. M. (1978). Racism and research: the case of the Tuskegee syphilis study. Hast. Cent. Rep. 8:21. doi: 10.2307/3561468 721302

[ref6] Brinkman L. Todorov A. Dotsch R. (2017). Visualising mental representations: a primer on noise-based reverse correlation in social psychology. Eur. Rev. Soc. Psychol. 28, 333–361. doi: 10.1080/10463283.2017.1381469

[ref7] Bronstein P. Farnsworth L. (1998). Gender differences in faculty experiences of interpersonal climate and processes for advancement. Res. High. Educ. 39, 557–585. doi: 10.1023/A:1018701722855

[ref8] Brown-Iannuzzi J. L. McKee S. Gervais W. M. (2018). Atheist horns and religious halos: mental representations of atheists and theists. J. Exp. Psychol. Gen. 147, 292–297. doi: 10.1037/xge0000376 29154618

[ref9] Bullynck M. Daylight E. G. De Mol L. (2015). Why did computer science make a hero out of Turing? Commun. ACM 58, 37–39. doi: 10.1145/2658985

[ref10] Cameron E. Z. White A. M. Gray M. E. (2016). Solving the productivity and impact puzzle: do men outperform women, or are metrics biased? Bioscience 66, 245–252. doi: 10.1093/biosci/biv173

[ref11] Campbell G. Denes R. Morrison C. (Eds.) (2000). Access denied: Race, ethnicity, and the scientific enterprise. New York, NY: Oxford University Press.

[ref12] Cocca C. (2014). The ‘broke Back test’: a quantitative and qualitative analysis of portrayals of women in mainstream superhero comics, 1993–2013. J. Graphic Novels Comics 5, 411–428. doi: 10.1080/21504857.2014.916327

[ref13] Cofnas N. Carl N. Woodley of Menie M. A (2018). Does activism in social science explain conservatives’ distrust of scientists? Am. J. Sociol. 49, 135–148. doi: 10.1007/s12108-017-9362-0

[ref14] Cone J. Brown-Iannuzzi J. L. Lei R. Dotsch R. (2021). Type I error is inflated in the two-phase reverse correlation procedure. Soc. Psychol. Personal. Sci. 12, 760–768. doi: 10.1177/1948550620938616

[ref15] Cuddy A. J. Fiske S. T. Glick P. (2008). Warmth and competence as universal dimensions of social perception: the stereotype content model and the BIAS map. Adv. Exp. Soc. Psychol. 40, 61–149. doi: 10.1016/S0065-2601(07)00002-0

[ref16] DeBruine L. (2018). Debruine/Webmorph: Beta release 2. Zenodo. doi: 10.5281/ZENODO.1162670

[ref17] Diego-Mas J. A. Alcaide-Marzal J. Poveda-Bautista R. (2022). Applying noise-based reverse correlation to relate consumer perception to product complex form features. Complexity 2022, 1–10. doi: 10.1155/2022/4641932

[ref18] Dixon R. M. Jones J. A. (2015). Conspiracist ideation as a predictor of climate-science rejection: an alternative analysis. Psychol. Sci. 26, 664–666. doi: 10.1177/0956797614566469 25814502 PMC4426137

[ref19] Dotsch R. Todorov A. (2012). Reverse correlating social face perception. Soc. Psychol. Personal. Sci. 3, 562–571. doi: 10.1177/1948550611430272

[ref20] Dotsch R. Wigboldus D. H. J. Langner O. van Knippenberg A. (2008). Ethnic out-group faces are biased in the prejudiced mind. Psychol. Sci. 19, 978–980. doi: 10.1111/j.1467-9280.2008.02186.x 19000205

[ref21] Douglas K. M. Sutton R. M. Cichocka A. (2017). The psychology of conspiracy theories. Curr. Dir. Psychol. Sci. 26, 538–542. doi: 10.1177/0963721417718261 29276345 PMC5724570

[ref22] Duch J. Zeng X. H. T. Sales-Pardo M. Radicchi F. Otis S. Woodruff T. K.. (2012). The possible role of resource requirements and academic career-choice risk on gender differences in publication rate and impact. PLoS One 7:e51332. doi: 10.1371/journal.pone.0051332 23251502 PMC3520933

[ref23] Dunham Y. Srinivasan M. Dotsch R. Barner D. (2014). Religion insulates ingroup evaluations: the development of intergroup attitudes in India. Dev. Sci. 17, 311–319. doi: 10.1111/desc.12105 24205988

[ref24] Evans J. H. Hargittai E. (2020). Who doesn’t trust Fauci? The public’s belief in the expertise and shared values of scientists in the COVID-19 pandemic. Socius 6:2378023120947337. doi: 10.1177/2378023120947337

[ref25] Fiske S. T. Dupree C. (2014). Gaining trust as well as respect in communicating to motivated audiences about science topics. Proc. Natl. Acad. Sci. 111, 13593–13597. doi: 10.1073/pnas.1317505111 25225372 PMC4183178

[ref26] Fradkin C. Weschenfelder G. V. Yunes M. A. M. (2016). Shared adversities of children and comic superheroes as resources for promoting resilience. Child Abuse Negl. 51, 407–415. doi: 10.1016/j.chiabu.2015.10.010 26560233

[ref27] Freeman J. B. Ambady N. (2009). Motions of the hand expose the partial and parallel activation of stereotypes. Psychol. Sci. 20, 1183–1188. doi: 10.1111/j.1467-9280.2009.02422.x 19686295

[ref28] Freeman J. B. Ambady N. (2014). “The dynamic interactive model of person construal: coordinating sensory and social processes” in Dual-process theories of the social mind. eds. Sherman J. W. Gawronski B. Trope Y.. (New York, NY: The Guilford Press), 235–248.

[ref29] Fujiwara Y. Velasco R. C. L. Jones L. K. Hite R. L. (2022). Competent and cold: a directed content analysis of warmth and competence dimensions to identify and categorise stereotypes of scientists portrayed in meme-based GIFs. Int. J. Sci. Educ. 44, 694–715. doi: 10.1080/09500693.2022.2050560

[ref30] Grundmann R. (2021). COVID and climate: similarities and differences. WIREs Clim. Change 12, 1–7. doi: 10.1002/wcc.737 PMC8646552 34899990

[ref31] Harriger J. A. Wick M. R. Mendez K. Barnett B. (2022). With great power comes great responsibility: a content analysis of masculinity themes in superhero movies. Psychol. Men Masculinities 23, 353–361. doi: 10.1037/men0000398

[ref32] Hehman E. Flake J. K. Freeman J. B. (2015). Static and dynamic facial cues differentially affect the consistency of social evaluations. Personal. Soc. Psychol. Bull. 41, 1123–1134. doi: 10.1177/0146167215591495 26089347

[ref33] Huang J. Gates A. J. Sinatra R. Barabási A.-L. (2020). Historical comparison of gender inequality in scientific careers across countries and disciplines. Proc. Natl. Acad. Sci. 117, 4609–4616. doi: 10.1073/pnas.1914221117 32071248 PMC7060730

[ref34] Hugenberg K. Bodenhausen G. V. (2003). Facing prejudice: implicit prejudice and the perception of facial threat. Psychol. Sci. 14, 640–643. doi: 10.1046/j.0956-7976.2003.psci_1478.x 14629699

[ref35] Imhoff R. Woelki J. Hanke S. Dotsch R. (2013). Warmth and competence in your face! Visual encoding of stereotype content. Front. Psychol. 4, 1–8. doi: 10.3389/fpsyg.2013.00386 23825468 PMC3695562

[ref36] Ito T. A. Urland G. R. (2003). Race and gender on the brain: Electrocortical measures of attention to the race and gender of multiply categorizable individuals. J. Pers. Soc. Psychol. 85, 616–626. doi: 10.1037/0022-3514.85.4.616 14561116

[ref37] Ito T. A. Urland G. R. (2005). The influence of processing objectives on the perception of faces: an ERP study of race and gender perception. Cogn. Affect. Behav. Neurosci. 5, 21–36. doi: 10.3758/CABN.5.1.21 15913005

[ref38] Kabat G. C. (2017). Taking distrust of science seriously: to overcome public distrust in science, scientists need to stop pretending that there is a scientific consensus on controversial issues when there is not. EMBO Rep. 18, 1052–1055. doi: 10.15252/embr.201744294 28559435 PMC5494502

[ref39] Kinsella E. L. Ritchie T. D. Igou E. R. (2015a). Lay perspectives on the social and psychological functions of heroes. Front. Psychol. 6, 1–12. doi: 10.3389/fpsyg.2015.00130 25741302 PMC4330705

[ref40] Kinsella E. L. Ritchie T. D. Igou E. R. (2015b). Zeroing in on heroes: a prototype analysis of hero features. J. Pers. Soc. Psychol. 108, 114–127. doi: 10.1037/a0038463 25603370

[ref41] Kossowska M. Szwed P. Czarnek G. (2021). Ideology shapes trust in scientists and attitudes towards vaccines during the COVID-19 pandemic. Group Process. Intergroup Relat. 24, 720–737. doi: 10.1177/13684302211001946

[ref42] Koswatta T. J. Wingenbach G. Leggette H. R. (2023). Factors influencing public perception of science. J. Appl. Commun. 106, 1–25. doi: 10.4148/1051-0834.2442

[ref43] Kulas P. (2018). Antyelitarna narracja współczesnej polskiej prawicy [the anti-elite narrative of the contemporary polish right-wing]. Przegląd Socjologii Jakościowej [Qualitative Sociology Review] 14, 14–39. doi: 10.18778/1733-8069.14.4.02

[ref44] Larivière V. Ni C. Gingras Y. Cronin B. Sugimoto C. R. (2013). Bibliometrics: global gender disparities in science. Nature 504, 211–213. doi: 10.1038/504211a 24350369

[ref45] Ley T. J. Hamilton B. H. (2008). The gender gap in NIH Grant applications. Science 322, 1472–1474. doi: 10.1126/science.1165878 19056961

[ref46] Lick D. Carpinella C. Preciado M. Spunt R. Johnson K. (2013). Reverse-correlating mental representations of sex-typed bodies: the effect of number of trials on image quality. Front. Psychol. 4, 1–9. doi: 10.3389/fpsyg.2013.00476 23908637 PMC3727110

[ref47] Locke S. (1999). Golem science and the public understanding of science: from deficit to dilemma. Public Underst. Sci. 8, 75–92. doi: 10.1088/0963-6625/8/2/301

[ref48] Ma D. S. Correll J. Wittenbrink B. (2015). The Chicago face database: a free stimulus set of faces and norming data. Behav. Res. Methods 47, 1122–1135. doi: 10.3758/s13428-014-0532-5 25582810

[ref49] Maister L. De Beukelaer S. Longo M. R. Tsakiris M. (2021). The self in the Mind’s eye: revealing how we truly see ourselves through reverse correlation. Psychol. Sci. 32, 1965–1978. doi: 10.1177/09567976211018618 34761992

[ref50] Malcom S. M. (2020). Katherine Johnson (1918–2020). Science 368:591. doi: 10.1126/science.abc1546 32381711

[ref51] Mangini M. C. Biederman I. (2004). Making the ineffable explicit: estimating the information employed for face classifications. Cogn. Sci. 28, 209–226. doi: 10.1207/s15516709cog2802_4

[ref52] Miller D. I. Nolla K. M. Eagly A. H. Uttal D. H. (2018). The development of Children’s gender-science stereotypes: a Meta-analysis of 5 decades of U.S. draw-A-scientist studies. Child Dev. 89, 1943–1955. doi: 10.1111/cdev.13039 29557555

[ref53] Mitchell M. McKinnon M. (2019). ‘Human’ or ‘objective’ faces of science? Gender stereotypes and the representation of scientists in the media. Public Underst. Sci. 28, 177–190. doi: 10.1177/0963662518801257 30247096

[ref54] Mouchetant-Rostaing Y. Giard M.-H. Bentin S. Aguera P.-E. Pernier J. (2000). Neurophysiological correlates of face gender processing in humans: ERP study of face gender processing in humans. Eur. J. Neurosci. 12, 303–310. doi: 10.1046/j.1460-9568.2000.00888.x 10651885

[ref55] Muller J. N. Moroco A. Loloi J. Portolese A. Wakefield B. H. King T. S.. (2020). Violence depicted in superhero-based films stratified by protagonist/antagonist and gender. Cureus. 12:e6843. doi: 10.7759/cureus.6843 32181080 PMC7053689

[ref56] National Science Board, National Science Foundation. (2021). The STEM labor force of today: Scientists, engineers and skilled technical workers. Science and engineering indicators 2022 (NSB-2021-2). Eds. C. Hamel, T. Gore, N. Gough, M. Goryn. Available online at: https://ncses.nsf.gov/pubs/nsb20212

[ref57] Newsom J. T. (2023). Longitudinal structural equation modeling: A comprehensive introduction. New York, NY: Routledge.

[ref58] Ojeda J. T. Silvia P. J. Cassidy B. S. (2022). Mental representations of sickness positively relate to adaptive health behaviors. Evol. Psychol. 20:147470492211094. doi: 10.1177/14747049221109452 PMC10355308 35790386

[ref59] Olivola C. Y. Sussman A. B. Tsetsos K. Kang O. E. Todorov A. (2012). Republicans prefer republican-looking leaders: political facial stereotypes predict candidate electoral success among right-leaning voters. Soc. Psychol. Personal. Sci. 3, 605–613. doi: 10.1177/1948550611432770

[ref60] Olivola C. Y. Todorov A. (2010). Elected in 100 milliseconds: appearance-based trait inferences and voting. J. Nonverbal Behav. 34, 83–110. doi: 10.1007/s10919-009-0082-1

[ref61] Peters H. P. (2013). Gap between science and media revisited: scientists as public communicators. Proc. Natl. Acad. Sci. 110, 14102–14109. doi: 10.1073/pnas.1212745110 23940312 PMC3752168

[ref62] Pittinsky T. L. (2015). America's crisis of faith in science. Science 348, 511–512. doi: 10.1126/science.348.6234.511-a 25931544

[ref63] Raihl Froese Lilian, "Framing Dr. Fauci: the portrayal of Dr. Anthony Fauci by fox news and CNN in the early COVID-19 lockdown" (2023). 726. Available online at:https://cedar.wwu.edu/wwu_honors/726

[ref64] Räty H. Snellman L. (1992). Does gender make any difference? Common-sense conceptions of intelligence. Soc. Behav. Personal. Int. J. 20, 23–34. doi: 10.2224/sbp.1992.20.1.23

[ref65] Rothman K. J. (2010). Curbing type I and type II errors. Eur. J. Epidemiol. 25, 223–224. doi: 10.1007/s10654-010-9437-5 20232112 PMC2850991

[ref66] Rule N. O. Ambady N. (2010). Democrats and republicans can be differentiated from their faces. PLoS One 5:e8733. doi: 10.1371/journal.pone.0008733 20090906 PMC2807452

[ref67] Rule N. O. Ambady N. (2011). Judgments of power from college yearbook photos and later career success. Soc. Psychol. Personal. Sci. 2, 154–158. doi: 10.1177/1948550610385473

[ref68] Rule N. O. Bjornsdottir R. T. Tskhay K. O. Ambady N. (2016). Subtle perceptions of male sexual orientation influence occupational opportunities. J. Appl. Psychol. 101, 1687–1704. doi: 10.1037/apl0000148 27559624

[ref69] Rutjens B. T. Heine S. J. Sutton R. M. van Harreveld F. (2018a). “Attitudes towards science” in Advances in experimental social psychology, vol. 57. Eds. J. M. Olson (Academic Press), 125–165. doi: 10.1016/bs.aesp.2017.08.001

[ref70] Rutjens B. T. Sutton R. M. van der Lee R. (2018b). Not all skepticism is equal: exploring the ideological antecedents of science acceptance and rejection. Personal. Soc. Psychol. Bull. 44, 384–405. doi: 10.1177/0146167217741314 PMC5810918 29191107

[ref71] Rutledge P. E. (2020). Trump, COVID-19, and the war on expertise. Am. Rev. Public Adm. 50, 505–511. doi: 10.1177/0275074020941683

[ref72] Salita J. T. (2015). Writing for lay audiences: a challenge for scientists. Med. Writ. 24, 183–189. doi: 10.1179/2047480615Z.000000000320

[ref73] Sanchez C. Dunning D. (2021). The anti-scientists bias: the role of feelings about scientists in COVID-19 attitudes and behaviors. J. Appl. Soc. Psychol. 51, 461–473. doi: 10.1111/jasp.12748 33821031 PMC8013646

[ref74] Schmidt B. (2015). Gendered language in teaching evaluations. Available online at:https://benschmidt.org/profGender/#%7B%22database%22%3A%22RMP%22%2C%22plotType%22%3A%22pointchart%22%2C%22method%22%3A%22return_json%22%2C%22search_limits%22%3A%7B%22word%22%3A%5B%22his%20kids%22%2C%22her%20kids%22%5D%2C%22department__id%22%3A%7B%22%24lte%22%3A25%7D%7D%2C%22aesthetic%22%3A%7B%22x%22%3A%22WordsPerMillion%22%2C%22y%22%3A%22department%22%2C%22color%22%3A%22gender%22%7D%2C%22counttype%22%3A%5B%22WordCount%22%2C%22TotalWords%22%5D%2C%22groups%22%3A%5B%22unigram%22%5D%2C%22testGroup%22%3A%22C%22%7D

[ref75] Shattuck S. Cheney I. (2020). Picture a scientist. Eds. N. Bedu. United States: Uprising.

[ref76] Stack S. (2004). Gender, children and research productivity. Res. High. Educ. 45, 891–920. doi: 10.1007/s11162-004-5953-z

[ref77] Sternberg R. J. (1985). Implicit theories of intelligence, creativity, and wisdom. J. Pers. Soc. Psychol. 49, 607–627. doi: 10.1037/0022-3514.49.3.607

[ref78] Sternberg R. J. Conway B. E. Ketron J. L. Bernstein M. (1981). People’s conceptions of intelligence. J. Pers. Soc. Psychol. 41, 37–55. doi: 10.1037/0022-3514.41.1.37

[ref79] Stilgoe J. Lock S. J. Wilsdon J. (2014). Why should we promote public engagement with science? Public Underst. Sci. 23, 4–15. doi: 10.1177/0963662513518154 24434705 PMC5753839

[ref80] Tanaka J. W. (2001). The entry point of face recognition: evidence for face expertise. J. Exp. Psychol. Gen. 130, 534–543. doi: 10.1037/0096-3445.130.3.534 11561926

[ref81] Todorov A. Mandisodza A. N. Goren A. Hall C. C. (2005). Inferences of competence from faces predict election outcomes. Science 308, 1623–1626. doi: 10.1126/science.1110589 15947187

[ref82] Tourangeau R. Yan T. (2007). Sensitive questions in surveys. Psychol. Bull. 133, 859–883. doi: 10.1037/0033-2909.133.5.859 17723033

[ref83] Valentine T. Darling S. Donnelly M. (2004). Why are average faces attractive? The effect of view and averageness on the attractiveness of female faces. Psychon. Bull. Rev. 11, 482–487. doi: 10.3758/BF03196599 15376799

[ref84] Van Dooren W. Noordegraaf M. (2020). Staging science: authoritativeness and fragility of models and measurement in the COVID-19 crisis. Public Adm. Rev. 80, 610–615. doi: 10.1111/puar.13219 32836435 PMC7272834

[ref85] Watts R. (2007). Women in science: A social and cultural history. Abingdon, UK: Routledge.

[ref86] Weingart P. Guenther L. (2016). Science communication and the issue of trust. J. Sci. Commun. 15:C01. doi: 10.22323/2.15050301

[ref87] Weingart P. Muhl C. Pansegrau P. (2003). Of power maniacs and unethical geniuses: science and scientists in fiction film. Public Underst. Sci. 12, 279–287. doi: 10.1177/0963662503123006

[ref88] Zebrowitz L. A. McDonald S. M. (1991). The impact of litigants' baby-facedness and attractiveness on adjudications in small claims courts. Law Hum. Behav. 15, 603–623.

