# Supplementary material for: Picture a scientist: classification images of scientists are perceived as White, male, and socially inept
Source: Front Psychol. 2025 Apr 30;16:1575123. doi: 10.3389/fpsyg.2025.1575123 (PMC12075299; doi:10.3389/fpsyg.2025.1575123)
Supplement: Supplementary file 1 [file Supplementary_file_1.docx]

**Appendix A: Mixed-Effects Linear Models**

This Appendix includes alternate analyses to those presented in the main article. For brevity, we will focus on the interaction effects which comprised the major analyses of the main article. All analyses were conducted in *R* 4.2.2. Mixed-effects linear models were conducted using the *lmer* package, while marginal means were calculated using the *emmeans* package.

**Demographic Traits**

We conducted a mixed-effects linear model. The model included two fixed effects: Classification Image (CI; Scientist vs. Hero vs. Genius vs. Person) and Trait (10 traits; see Table 1 in the main article). The model also included a random effect for Participant. We found a significant CI x Trait interaction (Wald *χ*^2^ (27) = 448.79, *p* < 0.001). We next calculated marginal means. All significant contrasts can be found in Table A1.

**Valenced Traits**

Similar to the demographic traits, a mixed-effects linear model was conducted. The model included two fixed effects: Classification Image (Scientist vs. Hero vs. Genius vs. Person) and Trait (19 traits; see Table 1). The model also included a random effect for Participant. We found a significant CI x Trait interaction (Wald *χ*^2^ (54) = 371.35, *p* < 0.001). We next calculated marginal means. All significant contrasts can be found in Table A2.

**Table A1**

*All significant contrasts comparing Classification Images on the valenced traits*

| Trait | Comparison | *b* | *SE* | *p*-value |
| --- | --- | --- | --- | --- |
| East Asian | Genius CI - Scientist CI | 0.41 | 0.09 | < 0.001 |
| Female | Genius CI - Person CI | -1.05 | 0.09 | < 0.001 |
| Female | Hero CI - Person CI | -0.87 | 0.09 | < 0.001 |
| Female | Person CI - Scientist CI | 1.09 | 0.09 | < 0.001 |
| Male | Genius CI - Person CI | 1.03 | 0.09 | < 0.001 |
| Male | Hero CI - Person CI | 0.92 | 0.09 | < 0.001 |
| Male | Person CI - Scientist CI | -1.06 | 0.09 | < 0.001 |
| White | Genius CI - Scientist CI | -0.35 | 0.09 | 0.02 |

**Table A2**

*All significant contrasts comparing Classification Images on the Valenced Traits*

| Trait | Comparison | *b* | *SE* | *p*-value |
| --- | --- | --- | --- | --- |
| Brilliant | Genius CI - Scientist CI | 0.34 | 0.07 | < 0.001 |
| Brilliant | Hero CI - Scientist CI | 0.29 | 0.07 | 0.03 |
| Brilliant | Person CI - Scientist CI | 0.29 | 0.07 | 0.02 |
| Charismatic | Hero CI - Scientist CI | 0.45 | 0.07 | < 0.001 |
| Charismatic | Person CI - Scientist CI | 0.30 | 0.07 | 0.01 |
| Chill | Genius CI - Scientist CI | 0.30 | 0.07 | 0.02 |
| Chill | Hero CI - Scientist CI | 0.49 | 0.07 | < 0.001 |
| Chill | Person CI - Scientist CI | 0.30 | 0.07 | 0.02 |
| Competent | Person CI - Scientist CI | 0.29 | 0.07 | 0.04 |
| Cool | Genius CI - Scientist CI | 0.37 | 0.07 | < 0.001 |
| Cool | Hero CI - Scientist CI | 0.35 | 0.07 | < 0.001 |
| Cool | Person CI - Scientist CI | 0.30 | 0.07 | 0.02 |
| Engaging | Genius CI - Scientist CI | 0.38 | 0.07 | < 0.001 |
| Engaging | Hero CI - Scientist CI | 0.54 | 0.07 | < 0.001 |
| Engaging | Person CI - Scientist CI | 0.38 | 0.07 | < 0.001 |
| Entertaining | Genius CI - Scientist CI | 0.29 | 0.07 | 0.03 |
| Entertaining | Hero CI - Scientist CI | 0.50 | 0.07 | < 0.001 |
| Funny | Genius CI - Hero CI | -0.32 | 0.07 | 0.004 |
| Funny | Hero CI - Person CI | 0.39 | 0.07 | < 0.001 |
| Funny | Hero CI - Scientist CI | 0.59 | 0.07 | < 0.001 |
| Intelligent | Person CI - Scientist CI | 0.32 | 0.07 | 0.005 |
| Mean | Hero CI - Scientist CI | -0.38 | 0.07 | < 0.001 |
| Smart | Person CI - Scientist CI | 0.30 | 0.07 | 0.02 |
| Strict | Hero CI - Scientist CI | -0.34 | 0.07 | < 0.001 |
| Sweet | Genius CI - Hero CI | -0.39 | 0.07 | < 0.001 |
| Sweet | Hero CI - Person CI | 0.28 | 0.07 | 0.04 |
| Sweet | Hero CI - Scientist CI | 0.46 | 0.07 | < 0.001 |

**Table A2**

*Continued*

| Trait | Comparison | *b* | *SE* | *p*-value |
| --- | --- | --- | --- | --- |
| Warm | Genius CI - Hero CI | -0.36 | 0.07 | < 0.001 |
| Warm | Hero CI - Person CI | 0.30 | 0.07 | 0.01 |
| Warm | Hero CI - Scientist CI | 0.55 | 0.07 | < 0.001 |
| Well-spoken | Person CI - Scientist CI | 0.295546559 | 0.069986429 | 0.02 |
